# Post-transcriptional regulation of cancer/testis antigen MAGEC2 expression by TRIM28 in tumor cells

**DOI:** 10.1186/s12885-018-4844-1

**Published:** 2018-10-11

**Authors:** Xiao Song, Chengli Guo, Yutian Zheng, Ying Wang, Zhongtian Jin, Yanhui Yin

**Affiliations:** 1 Department of Immunology, School of Basic Medical Sciences, Key Laboratory of Medical Immunology of Ministry of Health, Peking University, Beijing, 100191 China; 2 Department of Hepatobiliary Surgery, Peking University People’s Hospital, Beijing, 100044 China

**Keywords:** Cancer/testis antigen, MAGEC2, Regulation, TRIM28, Tumor cells

## Abstract

**Background:**

Cancer/testis antigen MAGEC2 (also known as HCA587) is highly expressed in a wide variety of tumors and plays an active role in promoting growth and metastasis of tumor cells. However, little is known for the regulation of MAGEC2 expression in cancer cells.

**Methods:**

Western blotting and quantitative RT-PCR were performed to analyze MAGEC2 expression. Co-immunoprecipitation assay was applied for detecting the endogenous interaction of MAGEC2 and TRIM28 in tumor cells. Overexpression and knockdown assays were used to examine the effects of TRIM28 on the expression of MAGEC2 protein. Immunohistochemistry (IHC) staining was performed in hepatocellular carcinoma patients to evaluate the association between the expression of MAGEC2 and TRIM28. Proteasome inhibitors MG132 or PS-341 and lysosome inhibitor Chloroquine (CQ) were used to inhibit proteasomal or lysosomal-mediated protein degradation respectively.

**Results:**

We demonstrate that MAGEC2 interacts with TRIM28 in melanoma cells and MAGEC2 expression in tumor cells depends on the expression of TRIM28. The expression level of MAGEC2 protein was significantly reduced when TRIM28 was depleted in tumor cells, and no changes were observed in MAGEC2 mRNA level. Furthermore, expression levels of MAGEC2 and TRIM28 are positively correlated in MAGEC2-positive human hepatocellular carcinoma tissues (*p* = 0.0011). Mechanistic studies indicate that the regulatory role of TRIM28 on MAGEC2 protein expression in tumor cells depends on proteasome-mediated pathway.

**Conclusions:**

Our findings show that TRIM28 is necessary for MAGEC2 expression in cancer cells, and TRIM28 may serve as a new potential target for immunotherapy of cancer.

**Electronic supplementary material:**

The online version of this article (10.1186/s12885-018-4844-1) contains supplementary material, which is available to authorized users.

## Background

Cancer/testis (CT) antigens are a group of genes whose expression is typically restricted to germ cells, but are aberrantly expressed in various kinds of human tumors [1–3]. Due to their highly restricted expression pattern, CT antigen has long been considered as an ideal target for immunotherapy [2–5]. Since the first CT antigen MAGEA1 was identified in 1991, more than 200 different CT genes, including the melanoma antigen (MAGE), G antigen (GAGE), and X chromosome antigen (XAGE) multigene families, have been discovered [1, 6, 7]. MAGEC2 (also known as HCA587), a member of MAGE family, is a CT antigen expressed in tumors of various histological types, including hepatocellular carcinoma, melanoma, lung cancer, bladder cancer and breast cancer etc. [8–11]. Accumulating evidence has indicated that MAGEC2 expression is associated with hallmarks of aggressive cancers. For example, expression of MAGEC2 in primary melanoma is a potential predictor of metastasis [12]; MAGEC2 expression in breast cancer is correlated with poor clinical prognosis [13]. Recent studies revealed the oncogenic properties of MAGEC2 in facilitating cancer cell viability, proliferation and metastasis [14–17]. However, little is known about the regulation of MAGEC2 expression in tumor cells except that it is a direct target of miR-874 [18].

TRIM28 (also known as KAP1, TIF1β) is a well known transcriptional co-repressor of kruppel-associated box zinc finger proteins (KRAB-ZFPs) [19–21], regulating multiple aspects of mammalian physiology [22–27]. Recent studies revealed the elevated TRIM28 expression in different types of tumors, and moreover, high levels of TRIM28 expression are associated with aggressive clinical features and poor prognosis in most types of cancers [28–32]. In this study, we found that expression of MAGEC2 protein in tumor cells depends on the expression of TRIM28, a reduction in the level of endogenous TRIM28 expression in melanoma cells resulting in significantly decreased expression of MAGEC2 protein. To our knowledge, this is the first time to report the role of TRIM28 in regulating the expression of cancer/testis antigen MAGEC2.

## Methods

### Cells culture and reagents

Human melanoma cell line A375 was purchased from ATCC (USA; ATCC® CRL-1619™), human pancreatic cancer cell line AsPC1 and lung cancer cell line A549 were from National Infrastructure of Cell Line Resource (China; Catalog #: 3111C0001CCC000214 and 3111C0001CCC000002, respectively). All these cells were maintained in DMEM (ATCC) supplemented with 10% FBS (Invitrogen, USA). Human melanoma cell line Hs 695 T was purchased from Cobioer Biosciences Co., LTD (China; Catalog #:CBP60320) and maintained in MEM (Gibco, USA) supplemented with 1× MEM NEAA (Gibco), 1 mM Sodium Pyruvate (Gibco) and 10% FBS (Invitrogen). All cell lines were cultured in a humidified atmosphere containing 5% CO2 at 37 °C and routinely checked for mycoplasma contamination. MG132, Chloroquine, and 5-aza-2-deoxycytidine (5-aza) were purchased from Sigma-aldrich (USA), PS-341 (Bortezomib) was from MedChemExpress (MCE, USA). MG132 and Chloroquine were used at 20 μM, PS-341 and 5-aza were used at 10 μM.

### Antibodies

Antibodies used in this study were as follows: anti-GAPDH (AP0066, Bioworld, USA), anti-β-actin (AP0060, Bioworld), anti-TRIM28 (#4124, CST, USA; MB0014, Bioworld), anti-MAGEA1 (BS1217, Bioworld), anti-MAGEA3/6 (TA800918, Origene, USA), anti-MAGEA10 (15295–1-AP, Proteintech, USA), anti-MAGEC3(21491–1-AP, Proteintech), anti-FLAG (M185–3, MBL, Japan), and anti-mouse or rabbit IgG (H + L) HRP Conjugate (W4021/W4011, Promega). Anti-MAGEC2 monoclonal antibody was provided by Prof. Boquan Jin [33].

### Plasmids, siRNAs and transfection

To generate pCL-FLAG-MAGEC2 expression vector, pRK-FLAG-MAGEC2 (constructed previously) [34] was digested with Hind III/Not I and the resulting fragment was cloned into pCL. To construct pCL-TRIM28, the full length TRIM28 was obtained by RT-PCR from mRNA of A375 cells and cloned into pCL vector using Not I/EcoR I restriction sites. The following siRNA sequences were used in this study. si-NC (CAAUUGAUACCGCAGAUGA), si-TRIM28-#1 (UGACCAAGAUCCAGAAGCA), si-TRIM28-#2 (CACTGAGGACTACAACCTT), si-TRIM28-#3 (GGAGAUGAUCCCUACUCAA), TRIM28-#4 (GCAUGAACCCCUUGUGCUG), TRIM28-#5 (GCGAUCUGGUUAUGUGCAA). All siRNA oligonulciotides were purchased from Ribobio (Guangzhou, China).

Plasmids were transfected with Lipofectamine 2000 (Invitrogen) for Hs 695 T and AsPC1 cells. siRNAs were transfected with jetPRIME (Polyplus-transfection, Strasbourg, France) for A375, Hs 695 T and A549 cells.

### RNA extraction and quantitative RT-PCR (qPCR)

Total RNA was extracted from cultured cells with Trizol reagent (Life Technologies) according to the manufacturer’s instructions. cDNA was synthesized using Reverse Transcription Kit (Promega). qPCR was performed using SYBR Green qPCR Master Mix (Promega). β-actin mRNA was used as an endogenous control to normalize for MAGEC2 mRNA expression. Primers used for MAGEC2, forward:5’-GGCCCTGAGGAAGAACTGAG-3′, reverse:5’-TGAGATCCAACAGGCCTTGAC-3′; for β-actin, forward:5’-GCGGGAAATCGTGCGTGACATT-3′, reverse: 5’-GATGGAGTTGAAGGTAGTTTCGTG-3′.

### Immunoprecipitation (IP) and immunoblotting

IP and immunoblotting experiments were performed as previously described [17]. Briefly, A375 or Hs 695 T cells were lysed with IP buffer (50 mM Tris-HCl at pH 7.5, 150 mM NaCl, 1 mM EDTA and 0.5% NP-40) containing protease inhibitors (Roche, USA), and the lysates were used for immunoprecipitation with appropriate antibodies as well as protein A-Sepharose (GE Healthcare, USA). The precipitants were separated by SDS-PAGE and immunoblotted with specific antibodies and secondary anti-mouse or anti-rabbit antibodies conjugated to horseradish peroxidase (Promega). Visualization was achieved with chemiluminescence.

### Immunohistochemistry

Fifty-three HCC patients who underwent hepatectomy in the Peking University People’s Hospital were enrolled in this study for immunohistochemical analysis. The experiment was approved by the Ethics Review Committee of Peking University of Health Science Center. Paraffin-embedded tissue sections were deparaffinized with xylene and rehydrated with a graded series of ethanol. Endogenous peroxidase was blocked with 3% H_2_O_2_. Antigen retrieval was performed in solution of 1 mM EDTA and 10 mM Tris (for MAGEC2 staining) or 0.01 M citric acid (for TRIM28 staining) at 95 °C for 15 min. Normal goat serum (5%) was then applied to block any nonspecific binding sites. Sections were incubated with anti-MAGEC2 or anti-TRIM28 antibodies (CST) at 4 °C overnight, followed by adding dextran carrying anti-rabbit & mouse IgG conjugated to HRP and positive staining was developed using the Dako REAL EnVision (USA) detection system. Images of stained sections were imported into Olymus CX31 digital microscope (Olymus, Japan) for quantifying stained cells. The immunohistochemical staining of MAGEC2 and TRIM28 was scored (H score) according to staining intensity and percentage of positive cells. Tumor staining intensity was graded on a scale from 0 (negative), 1 (weak), 2 (moderate) to 3 (strong) and each intensity category was scored a percentage of tumor cells ranging from 0 to 100 (the sum of the percentages adds up to 100). The percentage score was then multiplied by its intensity category to obtain a final *H*-score, ranging from 0 to 300 [35].

### Statistical analyses

All experiments were repeated at least three times with consistent results. Quantitative results are presented as mean ± SD (standard deviation). The *p*-values were calculated using a two-tailed Student’s t-test. Differences were considered statistically significant at *P* < 0.05.

## Results

### Endogenous MAGEC2 interacts with TRIM28 in melanoma cells

**Fig. 1:**
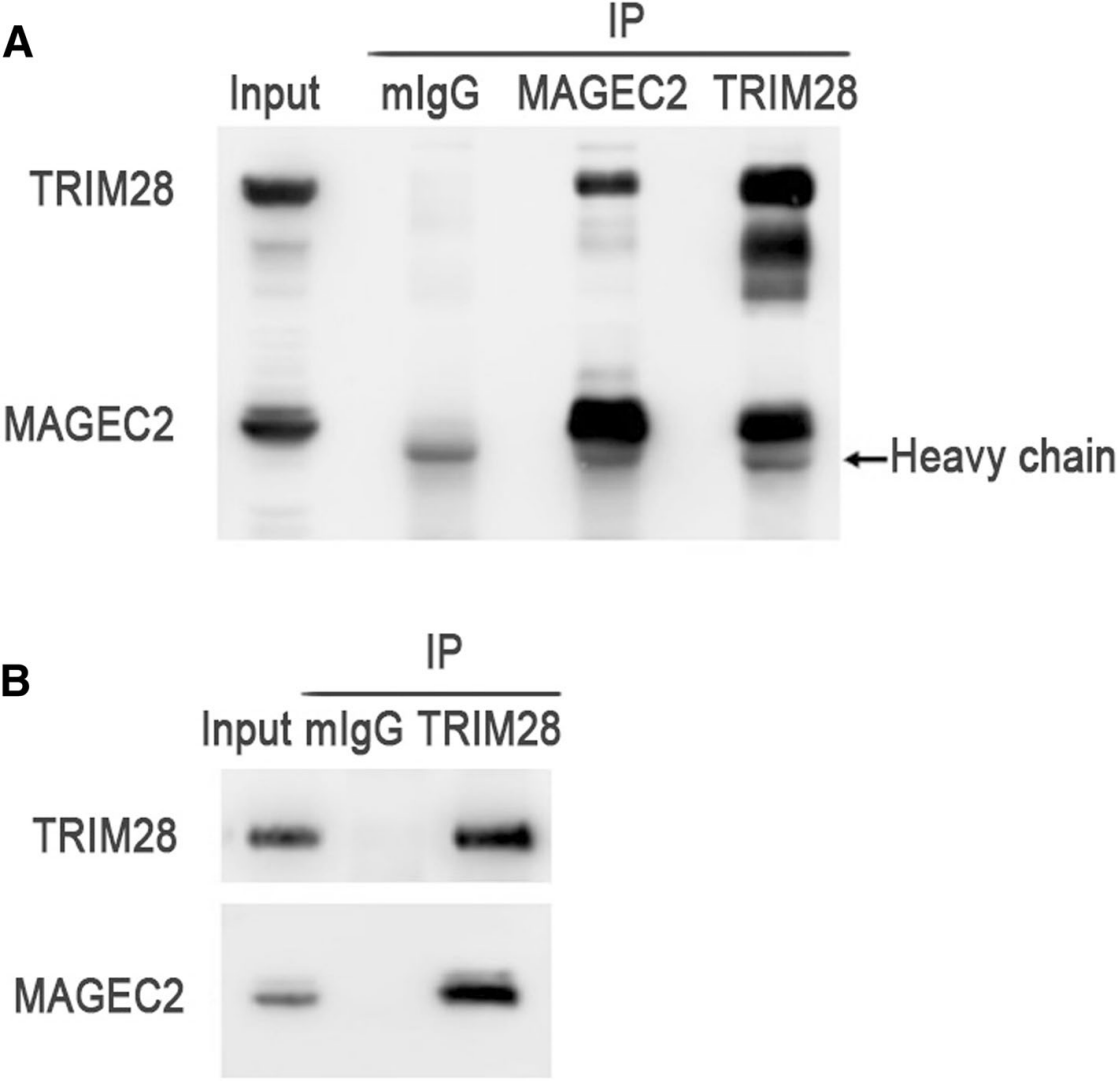
MAGEC2 interacts with TRIM28 in melanoma cell lines. **a** Endogenous MAGEC2 binds with TRIM28 in A375 cells. A375 cells (1 × 10^8^) were lysed and immunoprecipitated with mIgG, anti-MAGEC2 or anti-TRIM28 antibodies and immunoblotted with anti-MAGEC2 or anti-TRIM28 antibodies. Mouse IgG (mIgG) was used as a negative control for the immunoprecipitation. **b** Endogenous MAGEC2 binds with TRIM28 in Hs 695 T cells. Hs 695 T cells (1 × 10^8^) were lysed and immunoprecipitated with anti-mIgG or anti-TRIM28 antibodies and immunoblotted with anti-MAGEC2 or anti-TRIM28 antibodies. Mouse IgG (mIgG) was used as a negative control for the immunoprecipitation. The experiments were repeated at least three times

MAGEC2 has recently been reported to bind to TRIM28 in breast cancer cell lines HTB126 and HCC1806 [16]. To explore whether endogenous TRIM28 interacts with MAGEC2 in melanoma cells, co-immunoprecipitation experiments were performed using cell extracts obtained from human melanoma cells A375 which endogenously express both MAGEC2 and TRIM28 proteins. TRIM28 was found to be co-precipitated with MAGEC2, and alternatively, MAGEC2 was also detected in immunoprecipitates with anti-TRIM28 antibody (Fig. 1a). In addition, the interaction between endogenous MAGEC2 and TRIM28 was also detected in another human melanoma cell line Hs 695 T (Fig. 1b).

### Endogenous MAGEC2 protein expression in tumor cells depends on the existence of TRIM28 protein

**Fig. 2:**
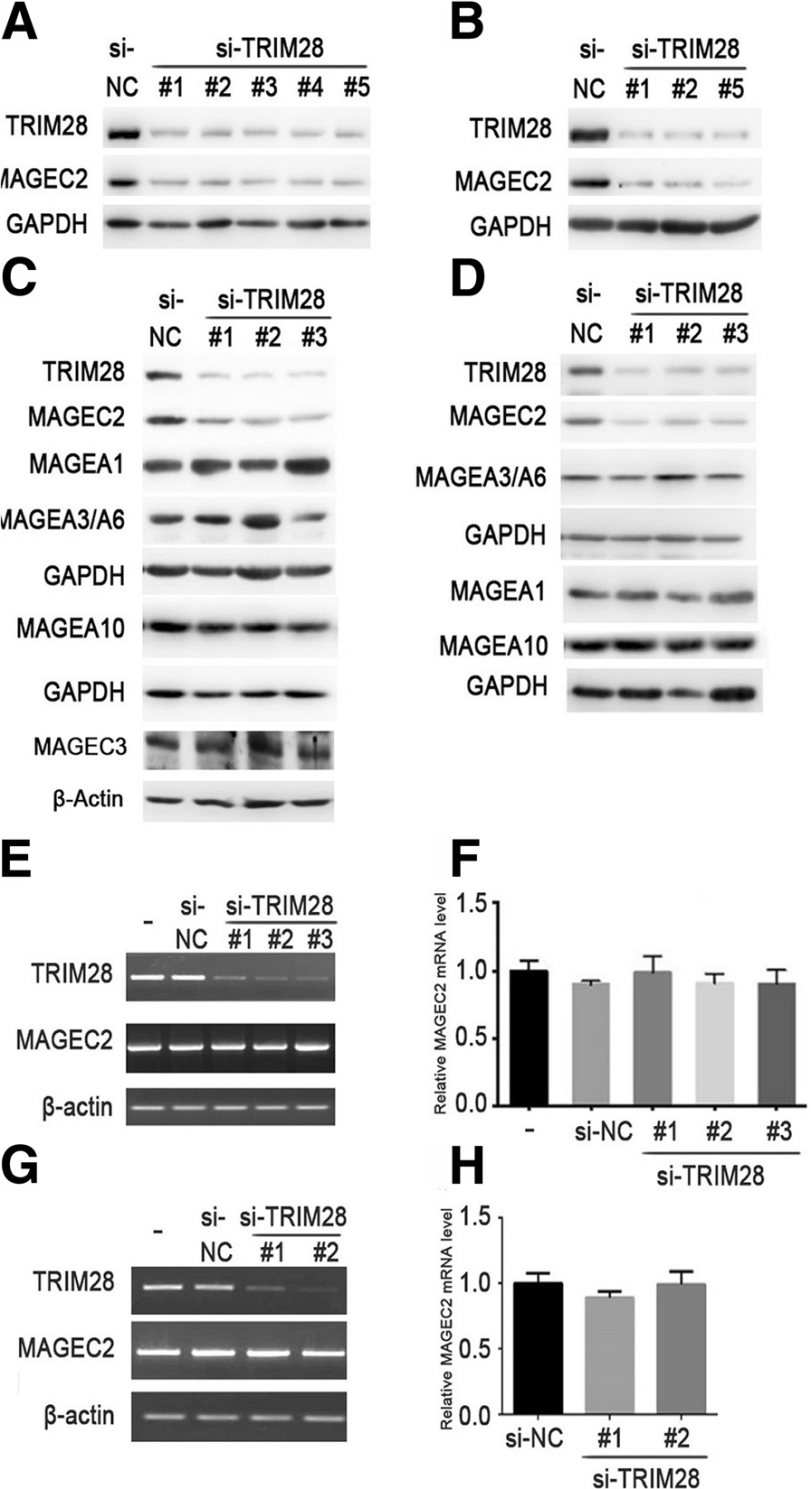
Knockdown of TRIM28 reduces MAGEC2 protein level and does not affect MAGEC2 mRNA level in melanoma cells. **a**, **b** Knockdown of TRIM28 decreases MAGEC2 protein level in A375 and Hs 695 T cells. TRIM28-specific siRNAs or control siRNA (si-NC) were transfected into A375 (**a**) or Hs 695 T cells (**b**) for 48 h, and cell lysates were immunoblotted with anti-MAGEC2 or anti-TRIM28 antibodies. Expression levels of GAPDH are indicated as an internal control. **c**, **d** Knockdown of TRIM28 does not affect the expression levels of other members of MAGE family in A375 cells (**c**) or Hs 695 T cells (**d**). A375 or Hs 695 T cells were transfected with TRIM28-specific siRNA for 48 h, and cell lysates were immunoblotted with indicated antibodies. **e**, **f**, **g**, **h** Knockdown of TRIM28 does not affect MAGEC2 mRNA level in A375 (**e**, **f**) or Hs 695 T cells (**g**, **h**). **e**, **f** Expression of MAGEC2 mRNA was examined in TRIM28-specific siRNA transfected-A375 cells by conventional PCR (E) or quantitative real-time PCR (**f**). **g**, **h** Knockdown of TRIM28 does not affect MAGEC2 mRNA level in Hs 695 T cells. Expression of MAGEC2 mRNA was examined in TRIM28-specific siRNA transfected-Hs 695 T cells by conventional PCR (**g**) or quantitative real-time PCR (**h**). Data are represented as mean ± SD. Each experiment was repeated three times

As TRIM28 has been reported to function as an E3 ubiquitin ligase [16], to determine whether MAGEC2 is a substrate of TRIM28 as E3 ligase, we evaluated the effects of TRIM28 on the expression of MAGEC2. Unexpectedly, we saw a dramatic decrease in MAGEC2 expression when endogenous expression of TRIM28 was silenced with small interfering RNA in both A375 and Hs 695 T cells (Fig. 2a and b). The observed effect was specific for MAGEC2 as TRIM28 silencing did not affect the levels of other members of MAGE family, such as MAGEA1, MAGEA3/6, MAGEA10 and MAGEC3 in A375 cells (Fig. 2c) and MAGEA1, MAGEA3/6 and MAGEA10 in Hs 695 T cells (Fig. 2d). MAGEC1 was not detected since it was not expressed in A375 cells as we previously reported [36]. To determine whether TRIM28 affects the mRNA level of MAGEC2, we performed conventional and quantitative real-time PCR in the presence or absence of TRIM28. The results showed that no significant changes in MAGEC2 mRNA level in TRIM28-depleted A375 cells (Fig. 2e and f) or Hs 695 T cells (Fig. 2g and h), suggesting that TRIM28 regulates MAGEC2 protein expression at post-transcriptional level.

### Exogenous and 5-aza induced expression of MAGEC2 protein is regulated by TRIM28 in tumor cells

**Fig. 3:**
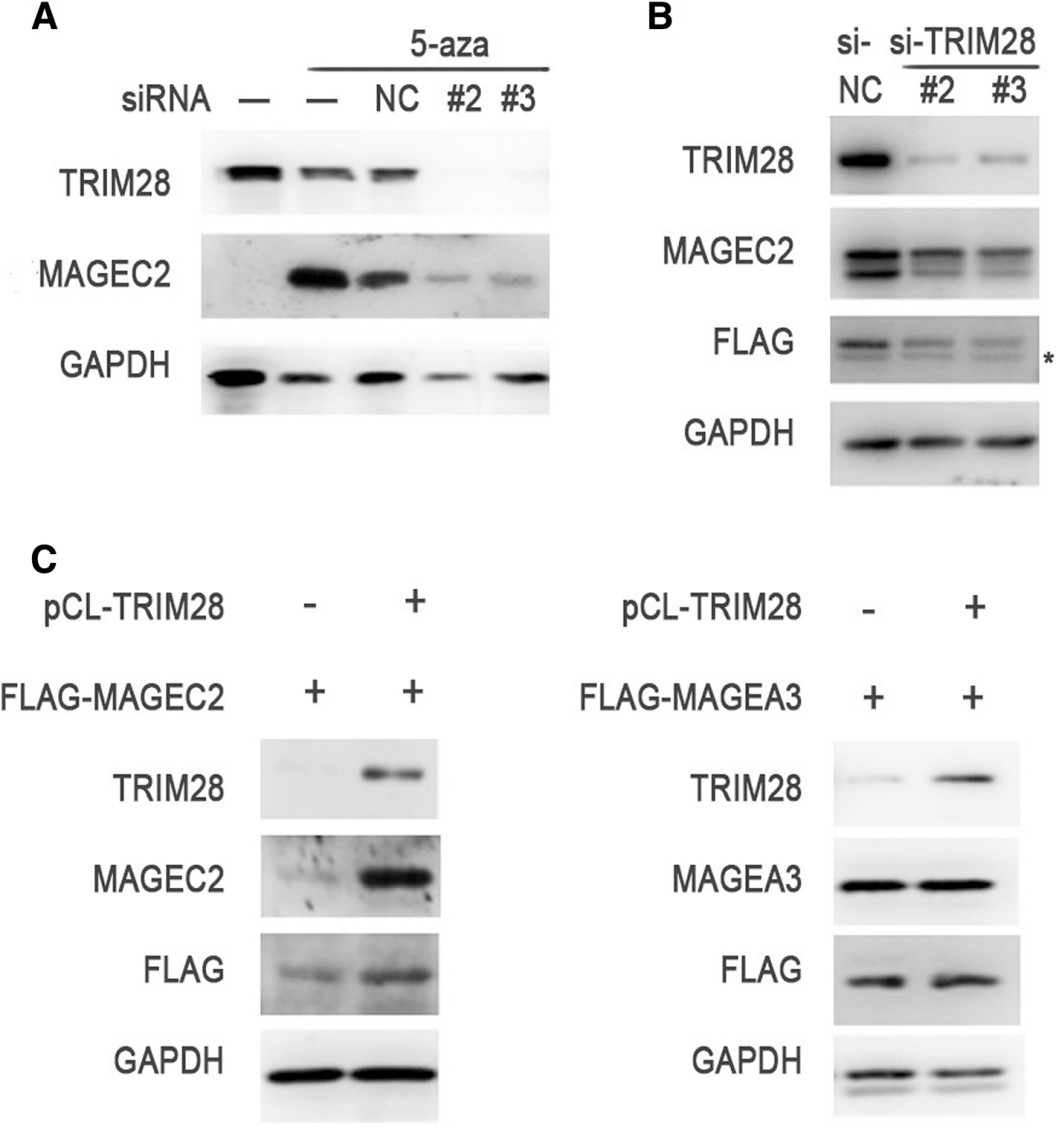
Induced or exogenous expression of MAGEC2 is regulated by TRIM28. **a** Knockdown of TRIM28 decreases the expression level of MAGEC2 induced by demethylating agent 5-aza in A549 cells. TRIM28 specific siRNAs or control (si-NC) were transfected into A549 cells for 12 h, followed by adding 5-aza (10 μM) for 4 days, and cell lysates were immunoblotted with anti-MAGEC2 or anti-TRIM28 antibodies. Expression levels of GAPDH are indicated as an internal control. **b** Knockdown of TRIM28 decreases the exogenous expression level of MAGEC2 in Hs 695 T cells. FLAG-tagged MAGEC2 expression vector and TRIM28 specific siRNAs or si-NC were co-transfected into Hs 695 T cells for 48 h, and cell lysates were immunoblotted with indicated antibodies. In MAGEC2 blots, the upper band represents FLAG-tagged MAGEC2 and the lower band represents endogenously expressed MAGEC2. *, unspecific bands. **c** Overexpression of TRIM28 increases exogenous MAGEC2 protein level in AsPC1 cells. pCL-TRIM28 and FLAG-MAGEC2 or FLAG-MAGEA3 (as a control) expression vectors were co-transfected into AsPC1 cells for 48 h, and cell lysates were immunoblotted with indicated antibodies. Each experiment was repeated three times

It was reported previously that the expression of MAGE family members can be induced by demethylation in MAGE-negative tumor cells [37]. To determine whether TRIM28 affects the induced expression of MAGEC2 in tumor cells, we treated MAGEC2-negative human lung cancer cells A549 with demethylating agent 5-aza-2-deoxycytidine (5-aza) in the presence or absence of TRIM28. Much reduced level of MAGEC2 was observed in TRIM28-knockdown A549 cells compared with control cells (Fig. 3a).

We further detect the effects of knockdown or overexpression of TRIM28 on the levels of exogenous expression of MAGEC2 protein in tumor cells. Hs 695 T cells were transfected with expression vector encoding FLAG-tagged MAGEC2 and TRIM28-specific siRNAs, and whole-cell extracts were prepared and immunoblotted with anti-MAGEC2 or anti-FLAG antibodies. As shown in Fig. 3b, both endogenous and exogenous MAGEC2 protein expression decreased significantly in TRIM28 knockdown cells. To test whether overexpression of TRIM28 affects the exogenous expression of MAGEC2, FLAG-tagged MAGEC2 or FLAG-tagged MAGEA3 and pCL-TRIM28 expression plasmids were co-transfected into human pancreatic cancer cell line AsPC1, which has no expression of MAGEC2 and much lower level of TRIM28 expression compared with that in A375 and Hs 695 T cells (Additional file 1: Figure S1). We observed that transfection with TRIM28 significantly increased the level of MAGEC2 protein expression (Fig. 3c, left panel), but not MAGEC2 mRNA level (Additional file 2: Figure S2), and no significant change was detected in MAGEA3 protein level in TRIM28-overexpression cells compared with control group (Fig. 3c, right panel).

### Expression level of MAGEC2 correlates with TRIM28 in MAGEC2-positive human tumor tissues

**Fig. 4:**
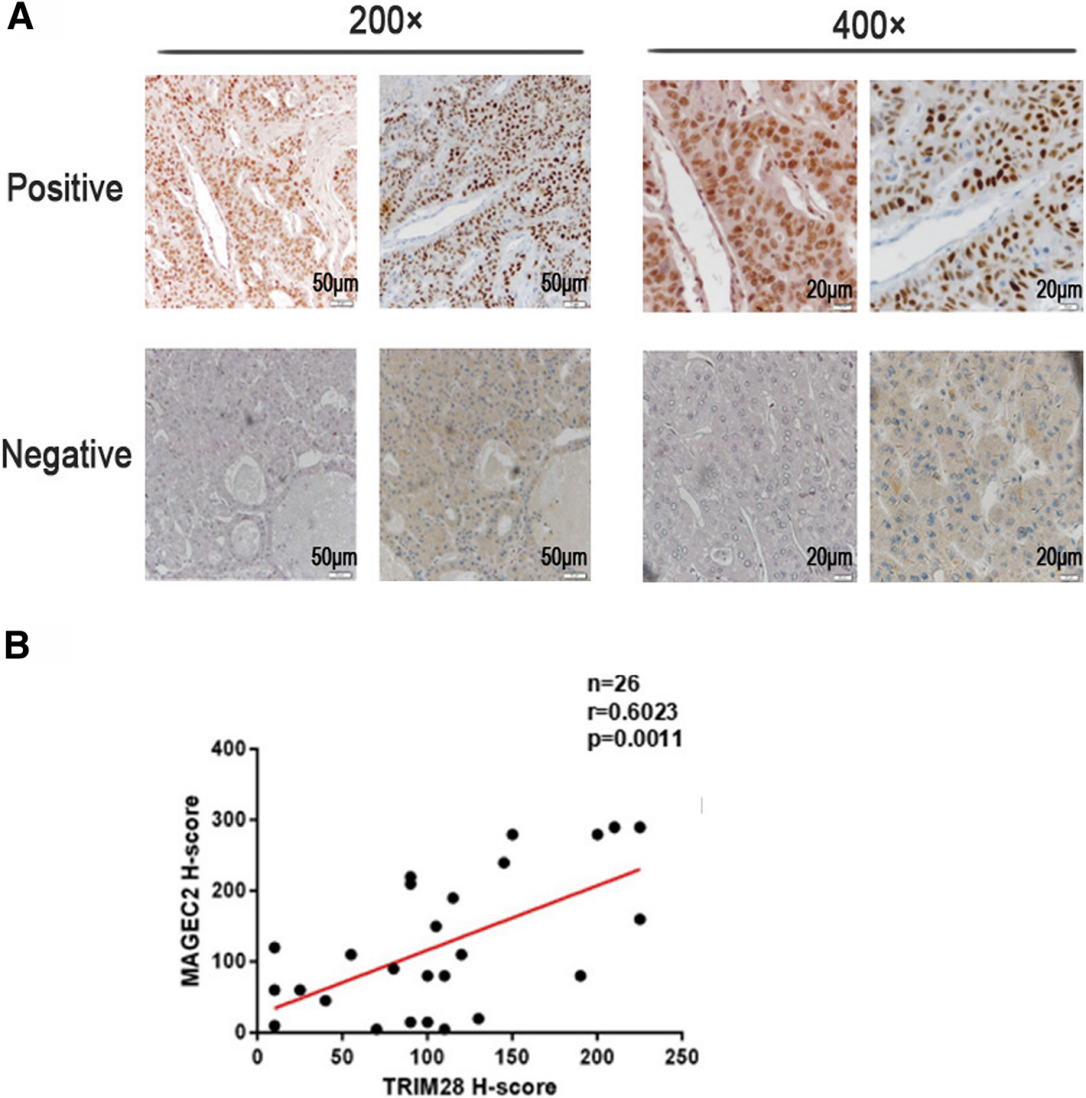
MAGEC2 expression correlates with TRIM28 in human hepatocellular carcinoma tissues. **a** The expression of MAGEC2 and TRIM28 in representative positive and negative samples. Scale (left, 200×), 50 μm; Scale (right, 400×), 20 μm. **b** Spearman correlation analysis of the expression levels of MAGEC2 and TRIM28 in MAGEC2-positive samples. *p* = 0.0011

We detected the expression of MAGEC2 and TRIM28 in human hepatocellular carcinoma (HCC) as MAGEC2 was reported to be more frequently overexpressed in HCC tissues [9, 38]. Serial sections were prepared from a total of 53 tissue samples, and immunohistochemically stained with anti-MAGEC2 and anti-TRIM28 antibodies. As shown in Fig. 4a, both proteins are distributed in the nucleus. MAGEC2 was detectable in 26/53 (42.4%) cases, while TRIM28 was observed in almost all tumor samples. On the basis of staining intensity and the percentage of positive cells, an H score was assigned to each sample for MAGEC2 and TRIM28, respectively. Spearman correlation test showed that the level of TRIM28 expression correlates positively with MAGEC2 expression in MAGEC2-positive samples (Fig. 4b).

### TRIM28 protects MAGEC2 protein from proteasomal degradation

**Fig. 5:**
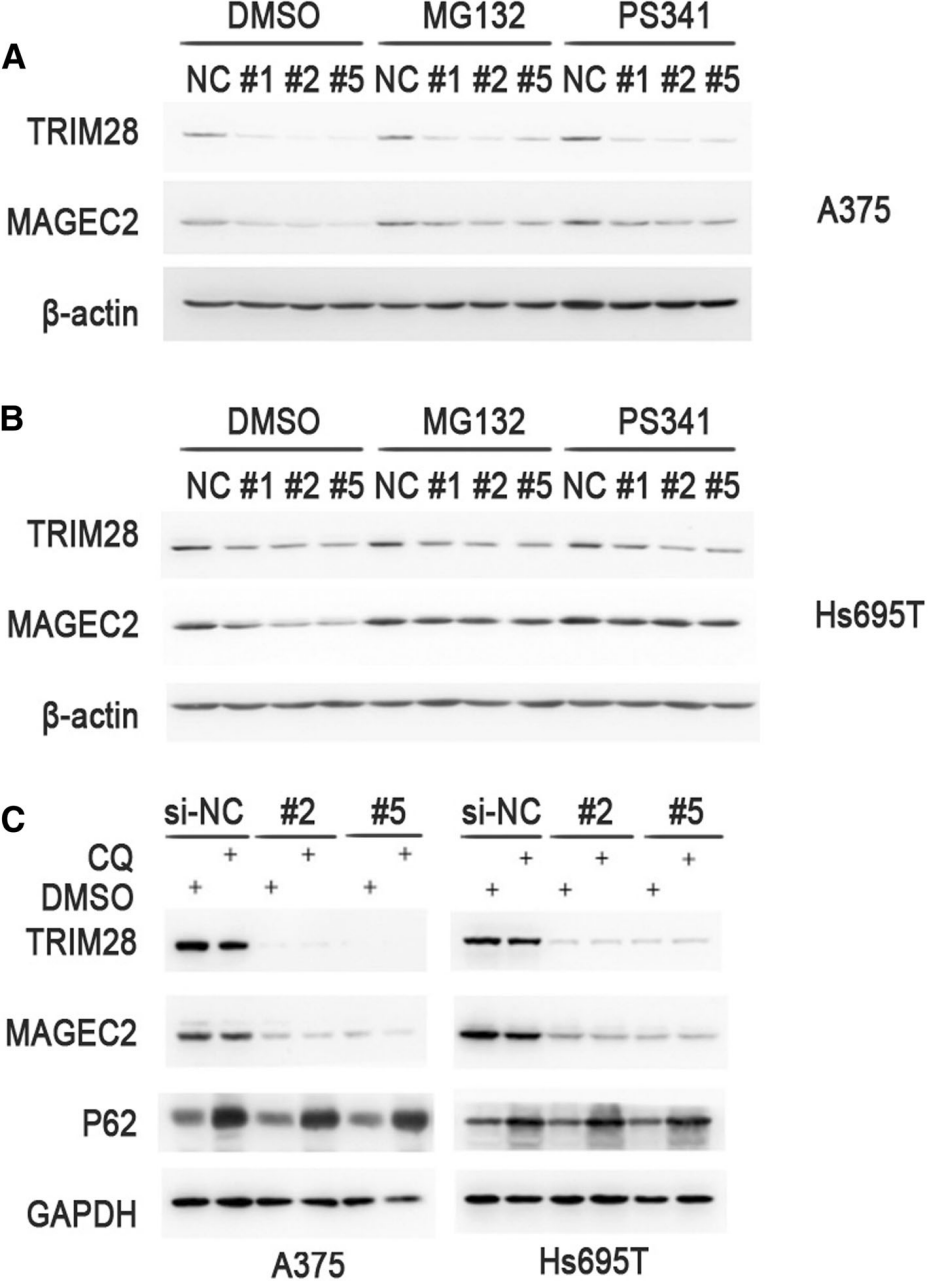
TRIM28 protects MAGEC2 from proteasomal degradation. **a**, **b** Downregulation of TRIM28 promotes the proteasome-mediated degradation of MAGEC2 in A375 cells (**a**) or Hs 695 T cells (**b**). TRIM28 specific siRNAs or si-NC were transfected into Hs695 T or A375 cells. Twenty-four hours after transfection, cells were treated with PS-341 (10 μM), MG132 (20 μM) or vehicle (DMSO) for 8 h before immunoblotting with indicated antibodies. **c** Lysosome inhibitor does not affect the level of MAGEC2 protein in TRIM28 knockdown Hs 695 T cells or A375 cells. TRIM28 specific siRNAs or si-NC was transfected into A375 (left panel) or Hs695 (right panel) T cells. Twenty-four hours after transfection, cells were treated with CQ (20 μM) or vehicle (DMSO) for 24 h before immunoblotting with indicated antibodies. Detection of p62 is served as a positive control for treatment with CQ. Each experiment was repeated at least three times

Proteasomal and lysosomal protein degradation represent the main pathway of intracellular protein degradation in eukaryotic cells [39]. To determine whether the influence of TRIM28 on MAGEC2 protein expression is related with proteasome-mediated protein degradation pathway, we analyzed MAGEC2 expression levels after treatment with the proteasome inhibitors MG132 or PS-341. We found that the reduction of MAGEC2 protein level in TRIM28-knockdown A375 cells could be inhibited by treatment with both MG132 and PS-341 (Fig. 5a), and similar result was also observed in Hs 695 T cells (Fig. 5b). In addition, we also detected the levels of MAGEC2 after treatment with lysosome inhibitor Chloroquine (CQ). No changes for MAGEC2 expression level were observed in TRIM28-knockdown Hs 695 T or A375 cells in the presence or absence of CQ, while the positive control p62 was accumulated greatly in all the groups treated with CQ (Fig. 5c). These results suggest that the regulation of TRIM28 on the level of MAGEC2 protein expression is proteasome-dependent.

## Discussion

As MAGEC2 is a cancer-testis antigen that is normally expressed in testis but aberrantly expressed in a broad spectrum of tumors, significant effort has been directed toward exploring its potential in tumor immunotherapy [40–43]. What controls or regulates the expression of MAGEC2 in cancer cells remains largely unknown to date. Here, we found that expression of MAGEC2 protein in tumor cells requires the existence of TRIM28. Depletion of TRIM28 expression in tumor cells not only reduced the endogenous expression level of MAGEC2 protein, but also decreased the exogenous expression of MAGEC2 protein.

One known mechanism for the control of MAGE gene expression is the regulatory role for methylation. Previously, Weber et al. reported that demethylating agent 5-aza-2-deoxycytidine was capable of inducing expression of MAGEA1, a member of MAGE family, in MAGEA1-negative melanoma cells [37]. We asked whether the expression of MAGEC2 was also under methylation control as the two proteins belong to the same family. MAGEC2-negative cell lines, human lung cancer cells A549 and pancreas cancer cells AsPC1, were treated with 5-aza, and the expression of MAGEC2 was induced in A549, but not in AsPC1 cells. However, when endogenous TRIM28 was depleted by TRIM28-specific siRNA, MAGEC2 expression could not be induced in A549 cells, suggesting that induced expression of MAGEC2 also depends on TRIM28 expression. Furthermore, we found that the expression level of MAGEC2 correlates positively with TRIM28 expression in MAGEC2-positive hepatocellular carcinoma tissues, while there was no significant difference for the average score of TRIM28 between MAGEC2-positive and MAGEC2-negative tumor tissues (Additional file 3: Figure S3), suggesting that existence of TRIM28 is a necessary but not a sufficient condition for the expression of MAGEC2 in tumor cells.

TRIM28, as a binding partner of members of the family of KRAB domain-containing zinc finger transcription factors, was considered as a critical transcriptional co-repressor and involved in diverse cellular processes such as development and differentiation of cells [19–21, 44]. To determine whether TRIM28 regulates MAGEC2 transcription, mRNA level of MAGEC2 was detected by conventional and quantitative real-time PCR in the presence or absence of TRIM28. The results showed that TRIM28 did not affect the level of MAGEC2 mRNA, implying that the regulatory role of TRIM28 on MAGEC2 expression is at the post-transcriptional level.

In addition to function as a classic transcriptional co-repressor, TRIM28, as a RING (really interesting new gene) domain protein, has been reported to act as an E3 ubiquitin ligase and multiple members of MAGE family bind with TRIM28 to facilitate efficient ubiquitin ligase activity of TRIM28. For example, MAGEC2 form complex with TRIM28 to promote TRIM28-dependent ubiquitination and degradation of tumor suppressor p53 in a proteasome-dependent manner in tumor cells [16]; MAGEA3/6 bind with TRIM28 to act as a cancer specific ubiquitin ligase and degrade AMPK tumor suppressor [45]. More recently, Addison J B et al. reported that TRIM28 binds directly with KRAB-ZNFs and protects KRAB-ZNFs from ubiquitin-dependent proteasomal degradation [28]. In this study, we also found that the regulatory role of TRIM28 on the expression of MAGEC2 protein in tumor cells is proteasome-dependent, which may be related with the E3 ubiquitin ligase activity of TRIM28.

MAGEC2 has been reported to increase phosphorylation of TRIM28-Ser824 induced by ataxia-telangiectasia-mutated (ATM) kinase, and enhance DNA damage repair [15]. To determine whether TRIM28 expression is regulated by MACEC2 expression in tumor cells, we also detected the expression level of TRIM28 in MAGEC2-knockdown melanoma cells and no change was observed (Additional file 4: Figure S4), suggesting that TRIM28 expression level is not regulated by MAGEC2.

## Conclusions

Our findings for the first time revealed that the expression of cancer testis antigen MAGEC2 in tumor cells depends on the existence of TRIM28, which is very important for further exploring the biological functions of MAGEC2 and TRIM28 in tumorigenesis and cancer development. In addition, as an important positive regulator of MAGEC2, TRIM28 might be a potential target for cancer therapy.

## Additional files

Additional file 1: **Figure S1**. Expression levels of TRIM28 and MAGEC2 are detected in different tumor cell lines. A375, Hs 695 T, and AsPC-1 cells were lysed and immunoblotted with anti-MAGEC2, anti-TRIM28 or anti-GAPDH antibodies. GAPDH was used as an internal control. (JPG 59 kb)

Additional file 2: **Figure S2**. Overexpression of TRIM28 does not affect exogenous MAGEC2 mRNA level in AsPC1 cells. pCL-TRIM28 and FLAG-MAGEC2 expression vectors were co-transfected into AsPC1 cells for 48 h, and mRNA expression of MAGEC2 and TRIM28 was examined by conventional PCR. GAPDH was used as an internal control. (JPG 76 kb)

Additional file 3: **Figure S3**. Distribution of TRIM28 expression level in MAGEC2-positive and MAGEC2-negative tissues of hepatocellular carcinoma patients. Human hepatocellular carcinoma tissues were immunohistochemically stained with anti-MAGEC2 or anti-TRIM28 antibodies, and H score was assigned to each sample for MAGEC2 and TRIM28, respectively. (JPG 55 kb)

Additional file 4: **Figure S4**. Knockdown of MAGEC2 does not affect TRIM28 expression. MAGEC2-specific siRNAs or control siRNA (si-NC) were transfected into A375 (A) or Hs 695 T cells (B) for 48 h, and cell lysates were immunoblotted with anti-MAGEC2 or anti-TRIM28 antibodies. Expression levels of β-actin are indicated as an internal control. (JPG 62 kb)
