# Supplementary figures and images for: Post-transcriptional regulation of cancer/testis antigen MAGEC2 expression by TRIM28 in tumor cells
Source: BMC Cancer. 2018 Oct 11;18:971. doi: 10.1186/s12885-018-4844-1 (PMC6182782; doi:10.1186/s12885-018-4844-1)

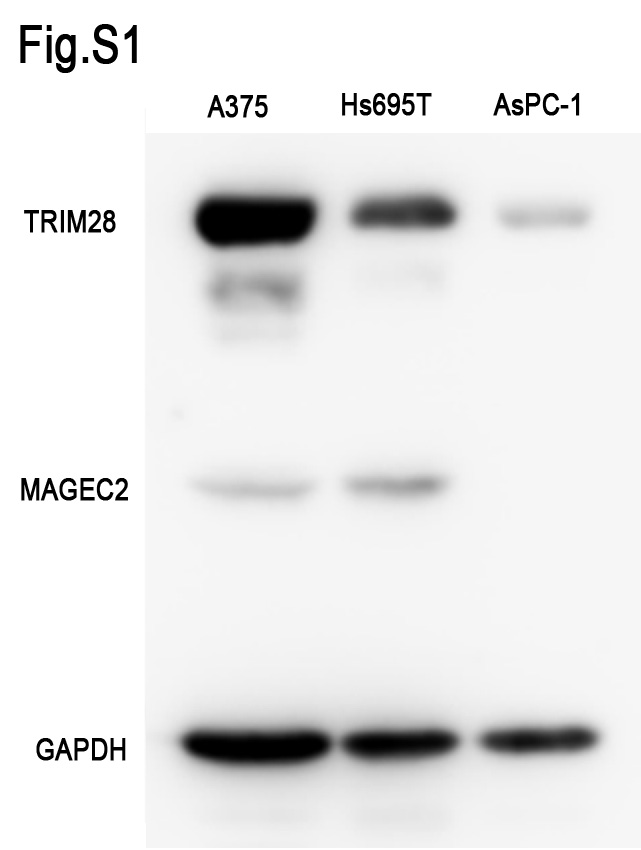

Supplement: Supplementary file 1 — Figure S1. Expression levels of TRIM28 and MAGEC2 are detected in different tumor cell lines. A375, Hs 695 T, and AsPC-1 cells were lysed and immunoblotted with anti-MAGEC2, anti-TRIM28 or anti-GAPDH antibodies. GAPDH was used as an internal control. (JPG 59 kb) [file 12885_2018_4844_MOESM1_ESM.jpg]

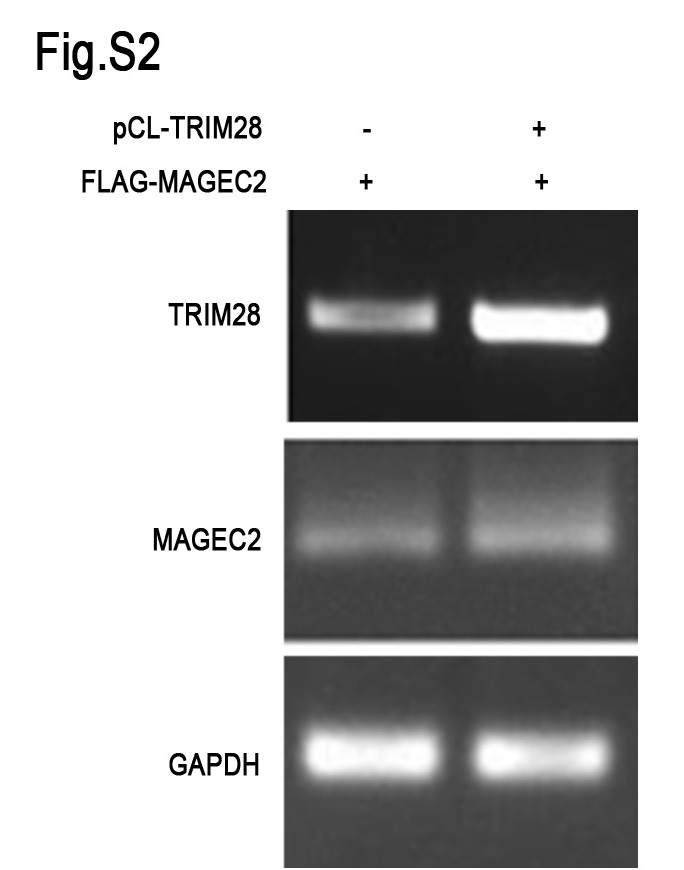

Supplement: Supplementary file 2 — Figure S2. Overexpression of TRIM28 does not affect exogenous MAGEC2 mRNA level in AsPC1 cells. pCL-TRIM28 and FLAG-MAGEC2 expression vectors were co-transfected into AsPC1 cells for 48 h, and mRNA expression of MAGEC2 and TRIM28 was examined by conventional PCR. GAPDH was used as an internal control. (JPG 76 kb) [file 12885_2018_4844_MOESM2_ESM.jpg]

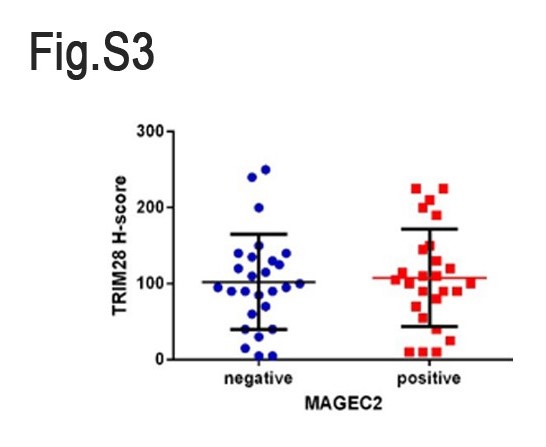

Supplement: Supplementary file 3 — Figure S3. Distribution of TRIM28 expression level in MAGEC2-positive and MAGEC2-negative tissues of hepatocellular carcinoma patients. Human hepatocellular carcinoma tissues were immunohistochemically stained with anti-MAGEC2 or anti-TRIM28 antibodies, and H score was assigned to each sample for MAGEC2 and TRIM28, respectively. (JPG 55 kb) [file 12885_2018_4844_MOESM3_ESM.jpg]

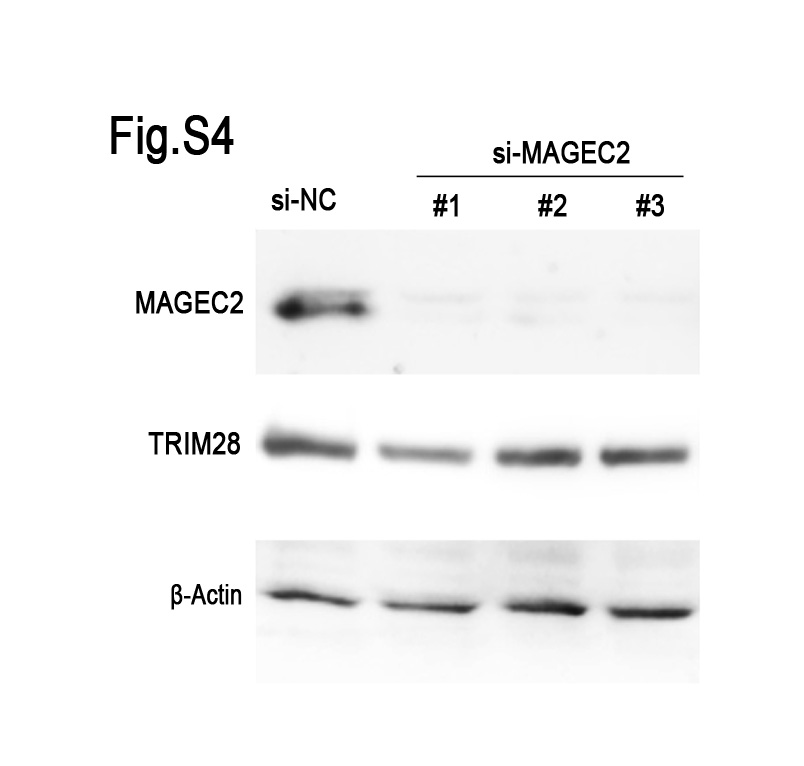

Supplement: Supplementary file 4 — Figure S4. Knockdown of MAGEC2 does not affect TRIM28 expression. MAGEC2-specific siRNAs or control siRNA (si-NC) were transfected into A375 (A) or Hs 695 T cells (B) for 48 h, and cell lysates were immunoblotted with anti-MAGEC2 or anti-TRIM28 antibodies. Expression levels of β-actin are indicated as an internal control. (JPG 62 kb) [file 12885_2018_4844_MOESM4_ESM.jpg]
